# Supplementary material for: Experience of switching from a daily to a less frequent administration of injection treatments
Source: PLoS One. 2022 Nov 30;17(11):e0278293. doi: 10.1371/journal.pone.0278293 (PMC9710744; doi:10.1371/journal.pone.0278293)
Supplement: S1 Dataset — (DOCX) [file pone.0278293.s002.docx]

**Minimal Underlying Data Set**

The published articles included in the analyses for this literature review are outlined below:

1. Johannsson G, Gordon MB, Højby Rasmussen M, Håkonsson IH, Karges W, Sværke C, et al. Once-weekly Somapacitan is Effective and Well Tolerated in Adults with GH Deficiency: A Randomized Phase 3 Trial. The Journal of Clinical Endocrinology & Metabolism. 2020;105(4):e1358-e76.
2. Johannsson G, Feldt-Rasmussen U, Hakonsson IH, Biering H, Rodien P, Tahara S, et al. Safety and convenience of once-weekly somapacitan in adult GH deficiency: a 26-week randomized, controlled trial. Eur J Endocrinol. 2018;178(5):491-9.
3. McNamara M, Turner-Bowker DM, Westhead H, Yaworsky A, Palladino A, Gross H, et al. Factors Driving Patient Preferences for Growth Hormone Deficiency (GHD) Injection Regimen and Injection Device Features: A Discrete Choice Experiment. Patient preference and adherence. 2020;14:781-93.
4. Humphriss E., Sheikh F. N., Seaman M., Ng D., Di Trapani K., W C. 016–Treatment Adherence to Somavaratan (VRS-317), a Long-Acting Growth Hormone for Children with Growth Hormone Deficiency (GHD): the VISTA Tria. Journal of pediatric nursing. 2017;34.
5. Qiao Q, Ouwens M, Grandy S, Johnsson KM, Kostev K. Adherence to GLP-1 receptor agonist therapy administered by once-daily or once-weekly injection in patients with type 2 diabetes in Germany. Diabetes, metabolic syndrome and obesity : targets and therapy. 2016;9:201-5.
6. Hauber AB, Nguyen H, Posner J, Kalsekar I, Ruggles J. A discrete-choice experiment to quantify patient preferences for frequency of glucagon-like peptide-1 receptor agonist injections in the treatment of type 2 diabetes. Curr Med Res Opin. 2016;32(2):251-62.
7. Zuurbier KWM, Reijs-Scheijgrond M, Visser LH. Dutch online survey patient reported outcomes (PROs) in MS: Improvement medication adherence after switch of daily glatiramer acetate 20mg/ml to 3x per week 40mg/ml. European Journal of Neurology. 2016;23(Suppl. 2):682.
8. Veneziano A, Cutter G, Al-Banna M, Rossi S, Zakharova M, Boyko A, et al. Higher medication satisfaction and treatment adherence in relapsing-remitting multiple sclerosis patients treated with glatiramer acetate 40 mg/ml three-times weekly compared with 20 mg/ml daily: 6-months results of the CONFIDENCE study. Multiple Sclerosis Journal. 2017;23(S3):641-2.
9. Cutter G, Veneziano A, Grinspan A, Al-Banna M, Boyko A, Zakharova M, et al. Satisfaction and adherence with glatiramer acetate 40mg/mL TIW in RRMS after 12 months, and the effect of switching from 20mg/mL QD. Multiple Sclerosis and Related Disorders. 2020;40:101957.
10. Osborne RH, Dalton A, Hertel J, Schrover R, Smith DK. Health-related quality of life advantage of long-acting injectable antipsychotic treatment for schizophrenia: a time trade-off study. Health Qual Life Outcomes. 2012;10:35.
11. Mathews M, Gopal S, Nuamah I, Hargarter L, Savitz AJ, Kim E, et al. Clinical relevance of paliperidone palmitate 3-monthly in treating schizophrenia. Neuropsychiatric disease and treatment. 2019;15:1365-79.
12. Cornford P, Jefferson K, Cole O, Gilbody J. Effects of Initiating or Switching to a Six-Monthly Triptorelin Formulation on Prostate Cancer Patient–Healthcare Interactions and Hospital Resource Use: a Real-World, Retrospective, Non-Interventional Study. Oncology and Therapy. 2018;6(2):173-87.
13. Khan A, Davda R, Dumas L, Payne H. Patient survey exploring preferences in frequency of administration of LHRH agonists. International Journal of CLinical Practice. 2012;66(Suppl. 176):1.
