# Supplementary material for: Experience of switching from a daily to a less frequent administration of injection treatments
Source: PLoS One. 2022 Nov 30;17(11):e0278293. doi: 10.1371/journal.pone.0278293 (PMC9710744; doi:10.1371/journal.pone.0278293)
Supplement: S1 Table — (PDF) [file pone.0278293.s003.pdf]

PICOS Framework for Systematic Reviews

- **Patient problem (or population):** Patients with any disease type receiving oral or injection treatment on a consistent treatment regimen (e.g., daily, weekly)
- **Intervention:** Oral or injection treatment of any type administered on a less frequent consistent regimen
- **Comparison or Control:** 'Comparison' between patients' experience of the original treatment regimen and the less frequent regimen as assessed by the outcomes listed below
- **Outcome:** The following outcomes were of interest in this research study: Adherence, Burden, Compliance, Convenience, Health-related quality of life, Preference, Safety, or Satisfaction
- **Study Type:** Regulated clinical trials or observational studies

Reference [manuscript ref. #]	P	I	C	O	S
Johannsson 2020 [24]	✓	✓	✓	✓	✓
Johannsson 2018 [25]	✓	✓	✓	✓	✓
McNamara 2020 [26]	✓	✓	✓	✓	✓
Humphriss 2017 [27]	✓	✓	✓	✓	✓
Qiao 2016 [16]	✓	✓	✓	✓	✓
Hauber 2016 [28]	✓	✓	✓	✓	✓
Zuurbier 2016 [29]	✓	✓	✓	✓	✓
Veneziano 2017 [30]	✓	✓	✓	✓	✓
Cutter 2020 [31]	✓	✓	✓	✓	✓
Osborne 2012 [32]	✓	✓	✓	✓	✓
Mathews 2019 [33]	✓	✓	✓	✓	✓
Cornford 2018 [34]	✓	✓	✓	✓	✓
Khan 2012 [35]	✓	✓	✓	✓	✓
