# Supplementary material for: Experience of switching from a daily to a less frequent administration of injection treatments
Source: PLoS One. 2022 Nov 30;17(11):e0278293. doi: 10.1371/journal.pone.0278293 (PMC9710744; doi:10.1371/journal.pone.0278293)
Supplement: Attachment — Submitted filename: Response to Reviewers (v1_0 - 2022.09.28).docx [file pone.0278293.s004.docx]

September 29, 2022	Adelphi Values

225 Franklin St., #1000

Boston, MA	02210

To whom it may concern:

Thank you again for the feedback provided by the academic editor and reviewers for our manuscript entitled *“**Experience of switching from a daily to a less frequent administration of injection treatments”* (PONE-D-21-40108).

We have reviewed the feedback from both the editor and reviewers, and our responses to the feedback related directly to the content of the manuscript and the associated tables, and figures are summarized in the table below this e-mail. In-text edits have been made within the manuscript in track-changes and, where appropriate, we have included reference to the line numbers where these edits have been made. We do hope that the revisions to the manuscript address the concerns of the reviewers, but if there is any further feedback, we are happy to revise further as needed. Please let us know.

One ask that related to supporting information associated with the manuscript was for the co-authors to revise our competing interest disclosures to specify who funded the study – and to indicate that these competing interests do not alter our adherence to the PLOS ONE policies on data sharing. This revised statement is included below the attached table with changes made in red text, as well as in the manuscript document itself.

Another point of consideration that was made by the editor following review was a request for our “minimal data set.” Based on the context of the study (i.e., a literature review) there is no traditional “data set.” However, we do provide the full list of articles, along with citations, as part of Table 2 should anybody wish to re-create our analysis.

As noted above, we hope the changes to the manuscript and the additional information provided improve the manuscript and allow it to be fit for publication in PLOS ONE. If there are any further questions or feedback surrounding this manuscript, please let us know!

Thank you again for the opportunity and for considering this manuscript for publication.

Best regards,

Andrew Yaworsky

[andrew.yaworsky@adelphivalues.com](mailto:Andrew.yaworsky@adelphivalues.com) | +1 (617) 720-0001

| Reviewer | Reviewer Feedback | Co-author response |
| --- | --- | --- |
| #1 | It is important to underline that childhood and adolescence are phases of possible fragility, in particular when it comes to illnesses, and that the impact of a greater appreciation of the method of administration could be reflected in multiple areas (psychological ... relational effects ..) | Thank you for this feedback. We have added text to the Discussion section to highlight the potential impact of treatment burden on patients’ lives during adolescence and childhood (lines 365-367). |
| #1 | Data for GHD are obtained from trials; we all know that the results of real medical practice are different from those of a study program. It should be considered that, after years of treatment, compliance could change regardless of the administration schedule.  In the discussion session only one comment should be added by the authors | Thank you for this feedback. This is a good point, and we have added a sentence related to compliance in a real world setting, and how this may change over time, within the Discussion section (lines 355-357). |
| #2 | It's unclear why the review focuses on the benefits of transition from daily to weekly GH regimens - the full range of patient experience seems more relevant. | Thank you for this feedback. The co-authors were initially interested in the transition from daily to weekly GH regimens as the daily regimen is currently the norm, and there are treatments in development that would allow for a less frequent, weekly regimen.  However, upon conducting the initial search and learning that there was a paucity of research in the area we decided to expand the search to include a broader range of experiences (e.g., different regimens and therapeutic regimens).  The lack of research in the area is highlighted in the Discussion section (lines 346-353). We have also added text to the Methods (lines 157-160) to further clarify. |
| #2 | The search strategy included two subsidiary searches for non-GH treatments. No information is provided on these search strategies - this is required before any judgement can be made on appropriateness. This is an important issue given the very wide diversity of possible inclusions / exclusions. | The additional two supplemental search strategies are described in two distinct sub-sections of the paper (*“Additional searches”*) in lines 155-172. They are also presented in Table 1. |
| #2 | The numbers of papers identified in the different search blocks isn't clear, particularly in 'Search 2'. Please clarify. | We have revised and re-formatted Figure 1 to better present the findings from each individual search. |
| #2 | The key challenge in this study is the absence of any quality measures in relation to the 13 studies reported. The diversity of methods, goals, illnesses and treatment modalities across such a small number of studies raises significant potential for methodological problems and the introduction of confounders, limiting comparability. Very little information is available on these issues in Table 2 and no standardised quality assessment tool is proposed. Please comment. | You are correct in stating that consistent assessment of the quality of the studies reviewed is a challenge given the variability in methodologies, therapeutic areas, etc. This was by virtue of the limited amount of data available.  That said, we utilized a PICOS framework to ensure that all studies included presented information related to the key research questions. We have added reference to this in the manuscript (lines 189-193).  We have also updated the Discussion to describe the limitation of reviewing articles with a range of methodologies, therapeutic areas, etc (lines 402-407). |
| #2 | Without a clear quality assessment and commentary, this isn't a normal systematic review and might best be presented as a pragmatic review. However, the assessment of quality is important given the challenges mentioned above and the utility of the study is limited without such insight. | Thank you for this feedback. We have revised the manuscript to reflect this as a pragmatic review (i.e., rather than systematic) throughout. |

**Revised competing interest disclosure statement (edits in red):**

JL and CLR are full-time employees of Pfizer, who funded the study. AY, MM, SS, and REL are employees of Adelphi Values LLC, who were paid consultants to Pfizer in connection with this research and development of this manuscript. DTB was an employee of Adelphi Values LLC at the time that the research was conducted. This does not alter co-author adherence to PLOS ONE policies on sharing data and materials.
